# Processing of sub- and supra-second intervals in the primate brain results from the calibration of neuronal oscillators via sensory, motor, and feedback processes

**DOI:** 10.3389/fpsyg.2014.00816

**Published:** 2014-08-01

**Authors:** Daya S. Gupta

**Affiliations:** Department of Biology, Camden County CollegeBlackwood, NJ, USA

**Keywords:** cerebellum, neuronal clock, polysensory processing, parietal, schizophrenia, interval timing

## Abstract

The processing of time intervals in the sub- to supra-second range by the brain is critical for the interaction of primates with their surroundings in activities, such as foraging and hunting. For an accurate processing of time intervals by the brain, representation of physical time within neuronal circuits is necessary. I propose that time dimension of the physical surrounding is represented in the brain by different types of neuronal oscillators, generating spikes or spike bursts at regular intervals. The proposed oscillators include the pacemaker neurons, tonic inputs, and synchronized excitation and inhibition of inter-connected neurons. Oscillators, which are built inside various circuits of brain, help to form modular clocks, processing time intervals or other temporal characteristics specific to functions of a circuit. Relative or absolute duration is represented within neuronal oscillators by “neural temporal unit,” defined as the interval between regularly occurring spikes or spike bursts. Oscillator output is processed to produce changes in activities of neurons, named frequency modulator neuron, wired within a separate module, represented by the rate of change in frequency, and frequency of activities, proposed to encode time intervals. Inbuilt oscillators are calibrated by (a) feedback processes, (b) input of time intervals resulting from rhythmic external sensory stimulation, and (c) synchronous effects of feedback processes and evoked sensory activity. A single active clock is proposed per circuit, which is calibrated by one or more mechanisms. Multiple calibration mechanisms, inbuilt oscillators, and the presence of modular connections prevent a complete loss of interval timing functions of the brain.

## INTRODUCTION

An important challenge for the modern neuroscience is to understand the mechanistic basis of the interval timing by the brain in the sub- and supra-second range (Wittmann, 1999; Lewis and Miall, 2003; Buhusi and Meck, 2005; Bueti et al., 2008; Coull et al., 2011). A steady output of the scientific literature over past several years has improved our understanding of the timing mechanisms. Old as well as new types of evidence have emerged, implicating multiple regions of the brain in the processing of time intervals (Harrington et al., 1998; Wittmann, 1999; Bueti et al., 2008; Coull et al., 2011; Parsons et al., 2013). Different regions involved in the perception of time intervals include the posterior parietal lobe, frontal lobe, insula, basal ganglia, and cerebellum (Harrington et al., 1998; Bueti et al., 2008; Coull et al., 2011; Teki et al., 2011a). It is generally agreed that multiple mechanisms are responsible for the measurement of time intervals by the brain (Lewis and Miall, 2003; Wittmann, 2009; Teki et al., 2011a). However, a consensus about the mechanisms has not emerged, which is clear from various models that have been proposed to explain the processing of various time intervals by the brain (Buonomano and Merzenich, 1995; Maass et al., 2002; Matell and Meck, 2004; Karmarkar and Buonomano, 2007; Wittmann, 2009; Teki et al., 2011a).

**FIGURE 1:**
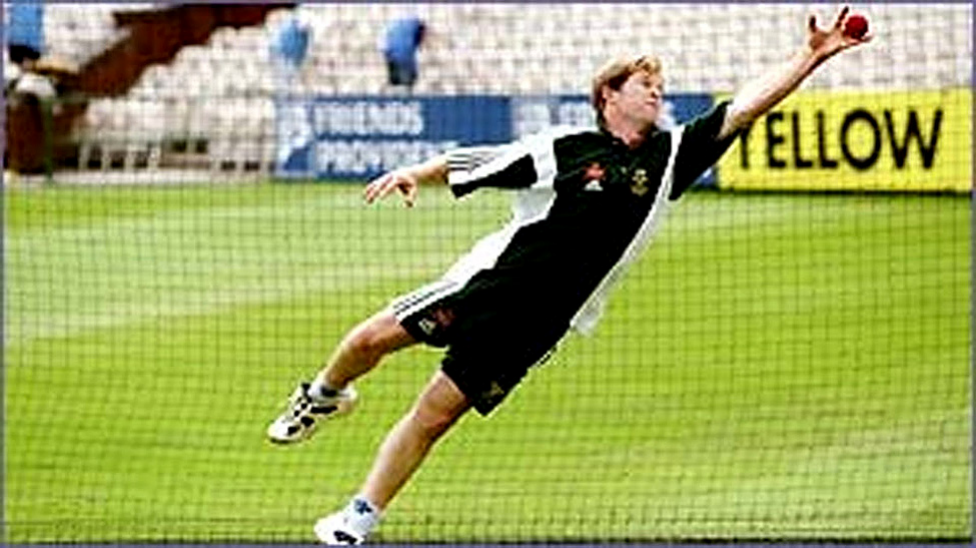
**The picture illustration of motor movements occurring over sub-second intervals.** A picture from a BBC website (http://news.bbc.co.uk/sportacademy/hi/sa/cricket/features/newsid_2653000/2653027.stm) shows a cricket player catching a ball in mid-air.The caption reads “Fielding is all about anticipation and quick thinking.” This caption underscores how motor actions taking place over few fractions of a second play a crucial role in the task of intercepting a cricket ball in a flight trajectory.

The processing of different time intervals plays an important role in different activities of the brain (Buhusi and Meck, 2005). The sub-second range timing (milliseconds) plays role in the motor control and the speech production (Buhusi and Meck, 2005). The supra-second range (seconds to minutes) interval timing plays role in foraging, decision making, and mental estimation of time, but it tends to be less accurate than the timing of sub-second intervals (Buhusi and Meck, 2005). In an example of a cricket fielder catching a ball during the mid-air trajectory (**Figure 1**), several time intervals are processed within the brain, both at the conscious and subconscious levels. The temporal intervals processed at a conscious level include anticipatory interval for the arrival of ball in the world-centric view and for executing motor movements to catch a ball, both in the supra-second range. Another time interval processed is the perception of the elapsed duration during an act of catching a ball (sub-to supra-second range). The subconscious processing of time intervals helps in the determination of speed of various motor movements, necessary for the successful execution of a task. For example, elapsed duration for the contraction of a muscle will determine the speed of a muscle contraction. Time intervals processed in muscle and joint movements are generally in the milliseconds range. Time intervals in above example are absolute, duration-based, which contrasts with the beat-based timing, which is relative to a temporal regularity, such as rhythmic beats (Teki et al., 2011a). It is shown that different networks in the brain are involved in both types of interval timing (Teki et al., 2011b).

In contrast to a much longer (24 h) circadian rhythm, which relies on the rhythmic expression of a set of clock genes with specific daily profiles in the suprachiasmatic nucleus and other parts of the brain (Rath et al., 2014), the interval timing in the sub-to supra-second range mainly relies on neuronal mechanisms. To understand the mechanisms underlying the neuronal clocks, one should first look at the mechanisms of the mechanical and electronic clocks. The mechanical and electronic clocks have inbuilt oscillators, which generate pulses corresponding to a standard unit of time. Both oscillators are based on a standard periodic event, such as the vibration of a quartz crystal or an oscillation of a pendulum. Based on the above analogy to the mechanical and electronic clocks, it is likely that the mechanism of neuronal clocks also includes an oscillator.

## OUTLINE OF THE PROPOSED NEURONAL CLOCK MECHANISM

**FIGURE 2:**
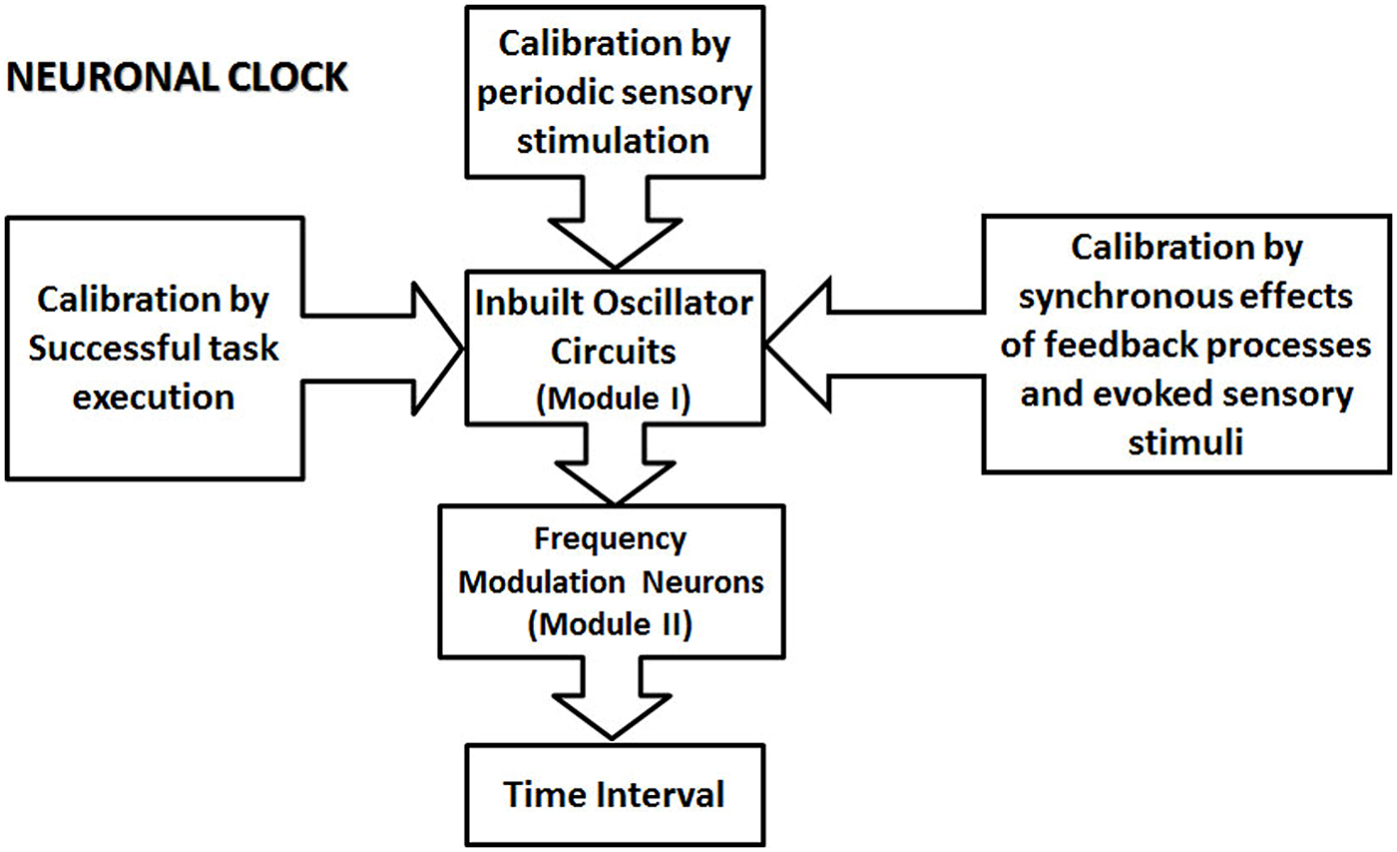
**The schematic illustration of the proposed modular neuronal clock.** A neuronal clock is formed by an inbuilt oscillator circuit (one per clock; Module I of the clock), which is calibrated by different types of mechanisms. Some other types of mechanism for proposed calibration are not indicated in this schematic. They include the basal ganglia circuit, and the premotor area, which contains representations of different learned movement patterns. The ability of different types of calibration mechanism to influence module I underscores the modular nature of the proposed clock model. The calibration of the clock mechanism involves the transfer of the physical time information from external events into the circuits in the central nervous system. The calibrated oscillator circuit activates a set of frequency modulator (FM) neurons in module II, resulting in the modulation of their activity. The state of the circuit, in module II, represented by the firing parameters, such as the frequency of the firing and the rate of the change of frequency encode various sub- and supra-second intervals.

**FIGURE 3:**
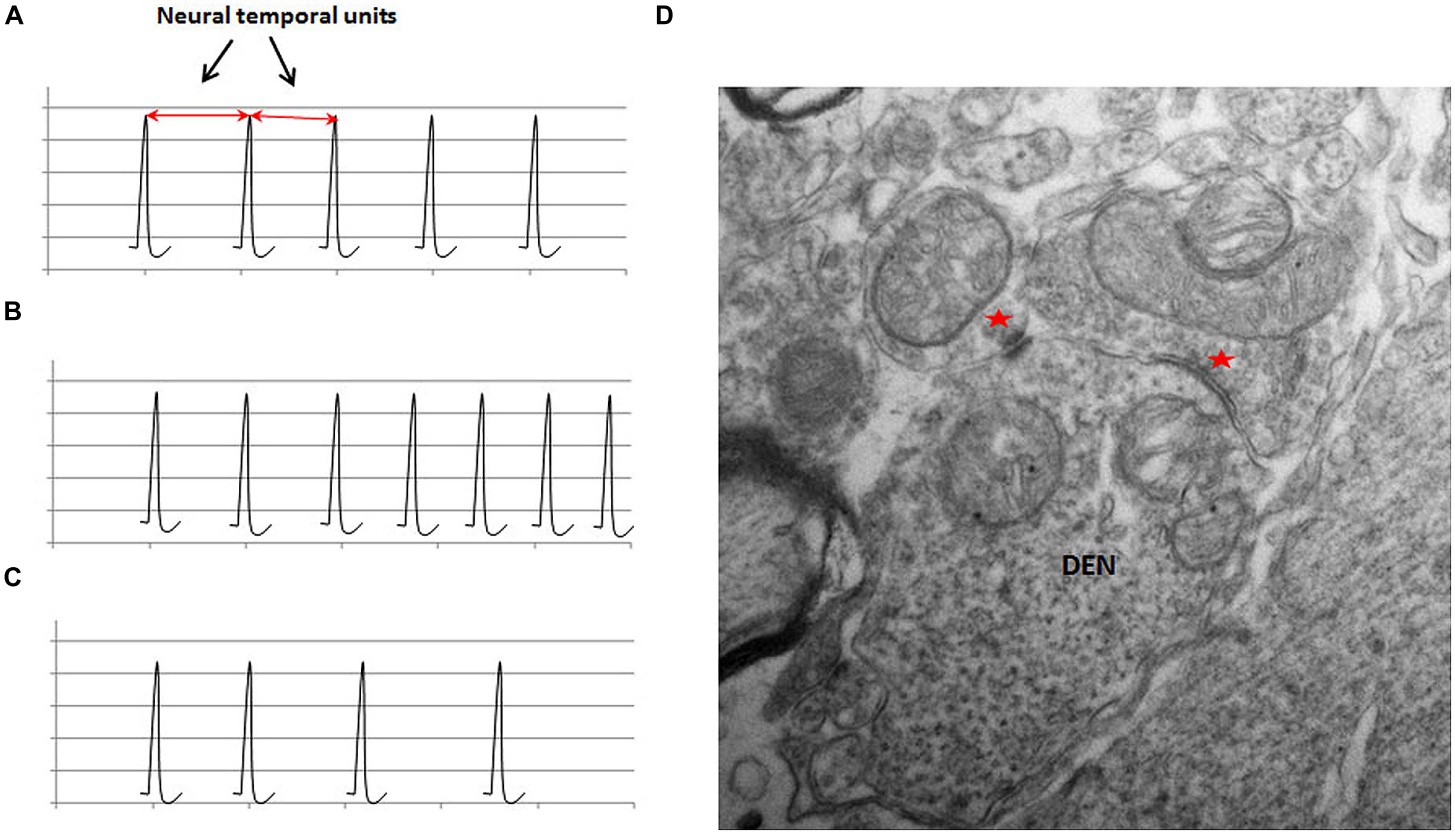
**The schematic illustration of the representation of physical time in neuronal circuits in **(A)**; the frequency modulation in (B) and **(C)**; and an ultrastructural basis of frequency modulation in **(D)****. The schematic in **(A)** shows the regular firing activity of a neuronal oscillator. The interval between two adjacent spikes during a regular activity is defined as the neural temporal unit, which is proposed to represent the physical time within the neuronal circuits. The value of the neural temporal unit is characteristically the same in a given circuit during a particular phase of a given activity. **(B)** Shows that the inter-spike interval progressively decreases in an FM neuron with ascending activity. **(C)** Shows the descending activity of an FM neuron. Note the progressive increase in the inter-spike interval. **(D)** An electron micrograph of a section from a rat spinal cord at a lower thoracic level (unpublished data from work done by the author at University of Louisville) shows a dendritic spine receiving inputs from two morphologically different synapses (indicated by red asterisks). Two different types of synapses can exhibit different types of plasticity in the same neuron, which can provide an ultrastructural basis for the modulation of the activity of neurons.

I propose a modular clock mechanism for the processing of the time interval in the sub- and supra-second ranges, which is based on the generic view of timing mechanism promoted by Ivry and Schlerf (2008), called “intrinsic models” (Ivry and Schlerf, 2008). Intrinsic models assume that “there is no specialized system for representing temporal information in the brain, asserting that time is inherent in neural dynamics” (Ivry and Schlerf, 2008). The proposed neuronal clock is formed by two main modules (see the schematic in **Figure 2**). The first module is formed by a neuronal oscillator. Neuronal oscillators generate temporally regular activity from the firing of the action potentials. I propose that the oscillators are built within different circuits across the brain, consistent with the intrinsic nature of timing mechanisms (Ivry and Schlerf, 2008). Temporal information is processed by the modular neuronal clocks, when the activity of the neuronal oscillator excites the proposed frequency modulator (FM) neurons (**Figure 3**) in the second module, which are endowed with special conductances, or are connected by synapses exhibiting plasticity. Special conductance properties, connections or even the nature of the input, such as recurrent excitation can produce modulation in the activity of FM neurons. The state of the circuit in the second module, determined by the frequency, or the rate of the change of frequency is proposed to code the time intervals in the brain.

Two important circuits, likely to contain the second module, forming neuronal clocks, include (i) the dorsal stream responsible for sensorimotor tasks, connecting the posterior parietal cortex and the motor, premotor and prefrontal areas of frontal cortex (Kaas et al., 2011), and (ii) cerebro-cerebellar loops that are responsible for the motor, and non-motor functions, for example working memory, executive tasks, and emotion (Strick et al., 2009; Bostan et al., 2013). The state of the circuit in the second module, following modulation in the activities of FM neurons, will code for various temporal parameters, such as the perception, reproduction and production of time intervals, and the speed of a motor movement.

The primary role of the oscillators in the modular clock mechanism is to represent the external physical time. In order to accurately represent the external physical time intervals, the oscillators are calibrated via tasks that involve a direct or indirect interaction with the external world. Since the oscillators are inbuilt, their intrinsic regular rhythm will be superimposed with fluctuations caused by feedback processes, as well as the sensory inputs, involved in the normal functioning of a circuit. In the next section, I will discuss (a) how the external time is represented by neuronal oscillators within circuits in the brain and (b) various types of regular neuronal activities that could potentially play role as the neuronal oscillators in the modular clocks.

## REPRESENTATION OF THE PHYSICAL TIME IN THE CENTRAL NERVOUS SYSTEM

The proposed neuronal clock mechanism outlined above incorporates inbuilt oscillators, which generate temporally regular activity representing a unit of time. Accordingly, a temporal unit interval, called neural temporal unit, between two adjacent spikes or bursts of spikes from the output of an oscillator, is proposed to encode the external physical time information required for processing time intervals in the sub-to supra-second range in the brain. Also note that the current model shares the requirement for an oscillator or a pulse generator with a pacemaker-accumulator model (Wittmann, 1999). However, there are significant differences between two models. An important difference is the inbuilt nature of the proposed oscillator circuit, which is in contrast to a separate temporal pacemaker in the pacemaker-accumulator clock model (Wittmann, 1999). The temporal pacemaker produces pulses with stable frequency, which is fed into an accumulator to record its number over a time period, and it is then compared with memory data (Wittmann, 1999). Furthermore, the inbuilt neuronal oscillators in the current proposal are subjected to fluctuations caused by the feedback processes and sensory inputs, which contrasts with the stable frequency of the temporal pacemaker proposed in the pacemaker-accumulator clock model (Wittmann, 1999).

Note that the neural temporal unit will not be represented by a single value in oscillators across the brain. Instead, the neural temporal units will be represented by different values in different oscillators. This will allow mechanistic flexibility for the interaction between separate clock mechanisms (see the discussion in Evidence Supporting the Role of Neural Temporal Units). A characteristic feature of the neural temporal unit is “intrinsic regularity” during a particular task. Note that the proposed intrinsic regularity is not the same as the precise regularity of mechanical clocks. Instead, it conforms to a behavior resulting from the intrinsic regularity, dependent on the single neuron properties. The regular behavior of the proposed neuronal oscillator is further expected to exhibit statistical properties, which is not discussed in the current manuscript.

According to the current anatomical and physiological data, the following classes of circuits are likely to provide the functionality of oscillators in the proposed modular clock.

**FIGURE 4:**
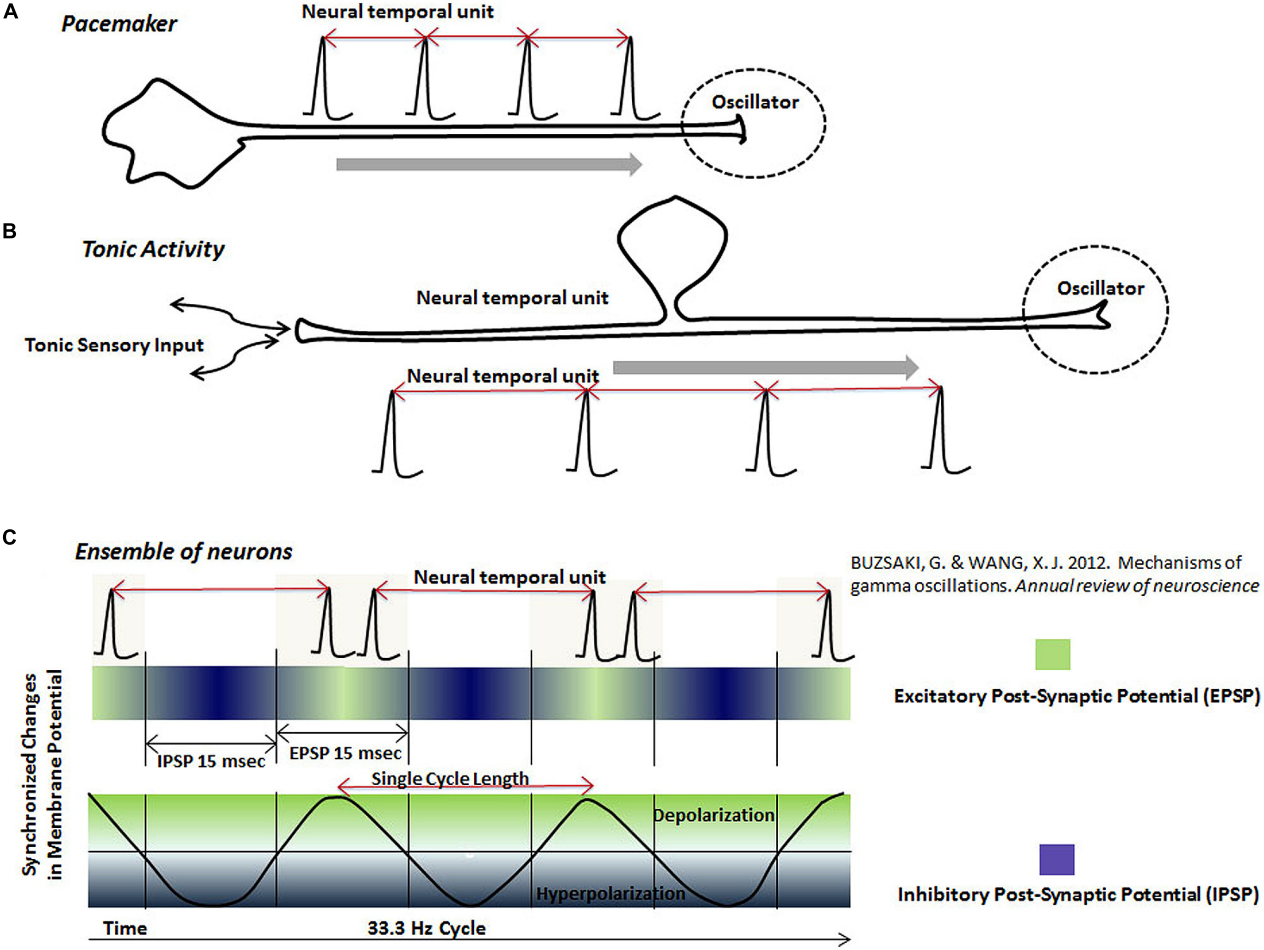
**The schematic representation of neural temporal units in various neuronal oscillators.** Neuronal oscillators are represented by spikes or spike bursts occurring at a regular interval. The interval between two adjacent spikes, called “neural temporal unit”, is proposed to represent the physical time in the brain. Different types of the proposed oscillator circuits are: **(A)** Pacemaker neurons, which generate spikes at a regular interval. The pacemaker neurons may project to other regions, where its output would serve as an oscillator. **(B)** Tonic rhythmic input into the brain, resulting from the tonic sensory stimulation, also provides the functionality of an oscillator. **(C)** This schematic shows that ensembles of neurons, connected together by the synchronized excitation and inhibition, fire bursts of spikes during the phase, when their membranes are most excitable (green). Firing of the bursts of spikes by neurons in an ensemble at a regular interval forms a neuronal oscillator mechanism in the brain. There are two types of information represented by the rhythmic activity of neurons in an ensemble: (i) The interval between two spike bursts or frequency of spike bursts (ii) Activity of individual neurons during each spike burst.

(a) Pacemaker neurons (**Figure 4A**): Pacemaker neurons have a common presence in the nervous system (Llinas, 1988). Regular rhythmic activity of pacemaker neurons is believed to emerge from special network connectivity and membrane conductances, such as a low threshold calcium conductance (Llinas, 1988). Suprachiasmatic nucleus, known for its role in the diurnal rhythm, contains pacemaker neurons (Schaap et al., 2003). Pacemaker activity is also observed *in vivo* and *in vitro* in deep cerebellar nuclei, which form the main output regions of the cerebellum (Jahnsen, 1986; Raman et al., 2000). Since the cerebellum plays an important role in cognitive measurements of time intervals (Lewis and Miall, 2003), it is likely that its pacemaker activity projects to form oscillators in other parts of the brain with which it has connections, for example, the posterior parietal cortex (Amino et al., 2001), also known to become co-active with the cerebellum in cognitive timing tasks (Lewis and Miall, 2003). In a separate example, dopamine neurons with slow pacemaker activity are present in the substantia pars compact (Grace and Onn, 1989; Kang and Kitai, 1993), which project to the medium spiny neurons of the striatum, forming synaptic connections near regions receiving excitatory inputs from the neocortex (Purves et al., 2001). The oscillator-type mechanism, resulting from the pacemaker activity of nigrostriatal dopamine neurons, is consistent with other types of evidence in favor of its role in timing mechanisms (Coull et al., 2011).

(b) Tonic sensory input (**Figure 4B**): Tonic activity of slowly adapting peripheral receptors projecting into brain could also serve as an oscillator. The highly sensitive Merkel’s disks (Purves et al., 2001), which are peripheral sensory receptors, are good candidates as a source of regular tonic input for various oscillator mechanisms. Tonic activity resulting from the stimulation of proprioceptors in tendons and muscles, project to the cerebellum and lateral intraparietal (LIP) area in the posterior parietal cortex (Prevosto et al., 2011), which could double up as an oscillator for clock mechanisms in circuits in the cerebellum and posterior parietal cortex. Role of the regular proprioceptive activity, acting as an oscillator, is likely to be combined with its role in motor movements in the cerebellar circuits.

(c) Regular synchronized activation and inhibition of ensemble of neurons (**Figure 4C**): Recording during specific tasks have shown ensembles of neurons undergoing synchronized changes of membrane potential, which are fleeting in both location and time (Kahana, 2006; Akam and Kullmann, 2010; Buschman et al., 2012; Buzsaki and Wang, 2012). These synchronized changes in membrane potential form the basis of dynamic electromagnetic gamma oscillations (10–50 Hz) detected in different parts of the brain (Kahana, 2006; Akam and Kullmann, 2010; Buschman et al., 2012; Buzsaki and Wang, 2012). It is further believed that synchronized changes in the membrane potential of ensemble of neurons represent excitatory and inhibitory post-synaptic changes (Sarko et al., 2013). Synchronization of the excitation and inhibition increases the chances of firing of neurons connected together in an ensemble during a particular phase of cycle (Fries et al., 2007), leading to bursts of spikes at regular intervals (Sarko et al., 2013; schematically represented in **Figure 4C**). The inevitable result of the synchronization of the activity of neurons connected together in ensembles is the temporal regularity of synchronized bursts. I propose that temporally regular bursts of spikes, generated by synchronized activation and inhibition of an ensemble of neurons during a cognitive task, could also function as an oscillator in a neuronal clock mechanism. Experimental evidence arguing this potential mechanism is provided by studies that had shown synchronous modulation of a large number of neurons in primate brains during various timing tasks (Durstewitz, 2004; Lebedev et al., 2008; Schneider and Ghose, 2012).

A key mechanistic requirement of a timing circuit is the ability to mark the onset and offset of a time interval. This could be achieved by incorporating a mechanism to start and stop a timer. Gamma oscillations are shown to be phase locked to or reset by different sensory stimuli (Eckhorn et al., 1988; Llinas et al., 1991; Joliot et al., 1994; Gray and Viana Di Prisco, 1997; Sannita, 2000; Fernandez-Ruiz and Herreras, 2013). Reset of an active synchronized ensemble of neurons by sensory stimuli would provide a mechanism to mark the onset or offset of time duration. Evidence suggesting this type of mechanism is provided by a study in monkeys involving self-timed saccading tasks, which showed that adjacent timing intervals were independent, consistent with the reset before the onset of each task (Schneider and Ghose, 2012). In this study, the reset of timing mechanism is believed to occur by internally generated cues (Schneider and Ghose, 2012).

Lebedev et al. (2008) have previously argued that distributed networks of neurons are responsible for the interval timing in a self-timed, delayed motor task, which was confirmed by the multielectrode recording from multiple areas of the brain in Macaque monkeys (Lebedev et al., 2008). They showed that the average rate of the change in frequency of neural activities obtained from simultaneous multi-electrode recording correlated with the delay period in a self-timed, delay motor task (Lebedev et al., 2008). The detection of the simultaneous modulation of neurons in multiple areas can be explained by their connection to a synchronized activity (**Figure 4C**). In addition to playing role as an oscillator, an ensemble of neurons with synchronized activity may also act as a feeder circuit between an oscillator and a circuit of FM neurons. Moreover, there are two types of information available for processing in ensembles of neurons with synchronized activities: (i) the interval between bursts and (ii) the activity of neurons during the bursts.

## EVIDENCE SUPPORTING THE ROLE OF NEURAL TEMPORAL UNITS IN THE INTERVAL TIMING

The processing of neural temporal units in neuronal circuits is also inferred from psychophysics experiments. In a task designed to compare the time intervals between two sound tones of a given frequency with a standard time interval, the benefits of training disappear when the standard time interval is changed (Wright et al., 1997). But the benefit of training is transferred when only the frequency of sound tones is altered (Wright et al., 1997). In this particular example, proposed oscillator activity for the clock mechanism is generated by the regular evoked bursts of action potentials in the brain after listening to regular beats of a given frequency. When the frequency of beats is changed, it would help to switch to a different proposed oscillator circuit, which would cause the benefits of the training to disappear. However, when the frequency of the tone was altered without changing the beat frequency, the same oscillator circuit would remain, continuing the benefits of the training. Thus, this study reveals an interval represented by regular beats, which plays role in timing tasks in above experiments, similar to the postulated neural temporal unit in the current model.

In another psychophysics experiment (Becker and Rasmussen, 2007), participants were first required to listen to an adapting rhythm of various beat frequencies. After listening to the adapting rhythm, participants listened to a fixed rhythm. After listening to the fixed rhythm, subjects were required to reproduce the fixed rhythm by a finger tapping task. An aftereffect of the adapting rhythm on the reproduction of the fixed rhythm was observed when the interval between adapting beats was closer to that of the fixed rhythm. This is explained by the proposed oscillator circuit, which is established by the longer lasting adapting rhythm with a periodicity closer to that of the fixed rhythm. In another result in the same study, the delivery of sound waves for the adapting and test rhythms from opposite ears produced similar aftereffects (Becker and Rasmussen, 2007), which indicates that there is a single neuronal clock mechanism for this particular task, localized to the right hemisphere, a likely assumption based on a past study (Harrington et al., 1998).

## THE PROCESSING OF TIME INTERVALS FROM THE “NEURAL TEMPORAL UNITS”

“Neural temporal unit,” defined as the interval between two adjacent regular spikes or spike bursts, (**Figure 3**) is proposed to represent the “dimension of time” in neuronal circuits processing various types of temporal information, such as the time interval, speed, and rate. Activity from the inbuilt oscillator is proposed to feed into the second module, where it produces modulations in the activity of FM neurons. The modulations in the activity of FM neurons help in coding different “time intervals” by generating changes in state-dependent parameters of the circuit in the second module, represented by the frequency change and the rate of the change of frequency or their combination.

Several past studies have demonstrated neurons undergoing modulation, with ascending or descending activity in the motor, premotor and parietal cortex during motor tasks, involving mental measurement of time intervals, and comparing lengths of time intervals (Leon and Shadlen, 2003; Durstewitz, 2004; Lebedev et al., 2008; Schneider and Ghose, 2012). Using simultaneous multi-electrode recordings, Lebedev et al. (2008) showed that the average rate of the change of the frequency of neurons with ascending activity correlates with the time intervals for motor tasks lasting 2.5–4.5 s (Lebedev et al., 2008). By recording activities of neurons with descending activity in LIP area, Schneider and Ghose (2012) showed that the production of inter-saccade interval in the range of 800 –1200 ms was positively correlated with the variations in the frequency, analyzed in 100 ms bins (Schneider and Ghose, 2012). Another study, by Mita et al. (2009), recorded activities in medial motor areas, in particular the pre-supplementary motor area in tasks requiring the minimum waiting periods of 2,4, and 8 s (Mita et al., 2009). They found neurons, with graded activity with exponential fit, during timing task, which showed selectivity to the duration of time intervals (Mita et al., 2009). These studies show that there are populations of neurons, undergoing modulations that are distributed across different parts of the brain, which have role in the processing of time intervals in the sub- to supra-second range. Further note that the modulated frequencies, detected in different studies involving timing tasks, range from 10 to 30 spikes/s (Lebedev et al., 2008; Schneider and Ghose, 2012), which is consistent with the connection of the neurons undergoing modulation with synchronized activities in the brain in the gamma range (20–40 Hz), believed to be important for information exchange (Buzsaki and Wang, 2012).

Short-term plasticity is one of several potential mechanisms, which can produce modulation in the activity of neurons in the central nervous system (Regehr, 2012). As depicted in **Figure 3**, neurons exhibiting short-term plasticity with excitation or depression, after excitation by the activity of oscillator circuits via synaptic inputs, can give rise, respectively, to an ascending (**Figure 3B**) or a descending activity (**Figure 3C**). Short-term plasticity, associated with facilitation or depression, has been demonstrated in mammalian cortical neurons (Gil et al., 1999). Theoretical studies have also shown that ascending and descending type of modulation in populations of neurons may be derived, respectively, from the properties of the network connections and the stochastic behavior of the neuronal populations (Gavornik and Shouval, 2011; Simen et al., 2011).

## READING THE ACTIVITY OF FM NEURONS

The activity of FM neuron is read by downstream circuits to give rise to time intervals required for various tasks and decision-making processes. A network of FM neurons in the second module of the proposed clock is under the influence of a single oscillator during a given task. The requirement for a single active oscillator circuit is based on the argument that multiple timing mechanisms for a single task or function will interfere with the homeostasis. The activity of FM neurons will be read by downstream circuits in order to produce temporal characteristics of a task, or a time interval for a delayed motor task, or production of a time interval. The proposed FM neurons are a mixed group of neurons with either ascending or descending activities. Both types of activities are likely to play role in various interval timing tasks. For example, a descending activity, directly or indirectly via a connection with an inhibitory interneuron, can produce the disinhibition. Disinhibition will occur when an inhibitory presynaptic neuron with a descending activity reaches a stage such that the post-synaptic neuron is not sufficiently inhibited. A disinhibition after a time interval encoded by the decay dynamics of an FM neuron can help in the activation of select neurons in a temporally specific manner. The activation of a motor unit by an FM neuron may help in a timed motor action. The activation of a post-synaptic neuron can also occur by an excitatory neuron with an ascending activity. Alternately, an excitation of a post-synaptic neuron with inhibitory activity can produce a timed-inhibition. The timed inhibition of a motor unit can inhibit activity of antagonist muscles, which can co-ordinate with activation of a synergistic muscle in timed motor actions.

Excitation of a post-synaptic neuron by another neuron with an ascending activity will produce behavior of a threshold oscillator (Borresen and Lynch, 2012). Theoretical considerations show that various logical operations, such as AND, OR, and NOT can be implemented by threshold oscillators with two inputs (Borresen and Lynch, 2012). Two logical states for neuronal circuits are “ON” state or the active state and “OFF” state or the inactive state of a neuron. The logical operations can help in determining the ON and OFF states of different neurons, which may control motor units or other neurons in the nervous system, helping in the decision-making processes. Together the decaying and ramping dynamics, and the logical operations would explain the timed motor behavior in animal experiments. Logical operations will also play a role in the muscle coordination by determining the activation (ON state) of synergistic muscles or the inhibition (OFF state) of antagonistic muscles.

## CALIBRATION OF THE PROPOSED MODULAR CLOCK MECHANISM

An important question that is not adequately addressed by various models is how neuronal clocks are calibrated. The accuracy of neuronal clocks will depend on how closely time intervals measured reflect time intervals of the external physical events. Various abnormalities of the central nervous system functions will result from the lack of calibration of neuronal clocks due to the inability to meet specific temporal requirements of external tasks. It is now believed that the timing intervals for motor and sensory tasks are calibrated by feedback mechanisms (Parsons et al., 2013). But how it is done is not well understood (Parsons et al., 2013).

## ROLE OF MOTOR PROCESSES IN THE CALIBRATION MECHANISM

**FIGURE 5:**
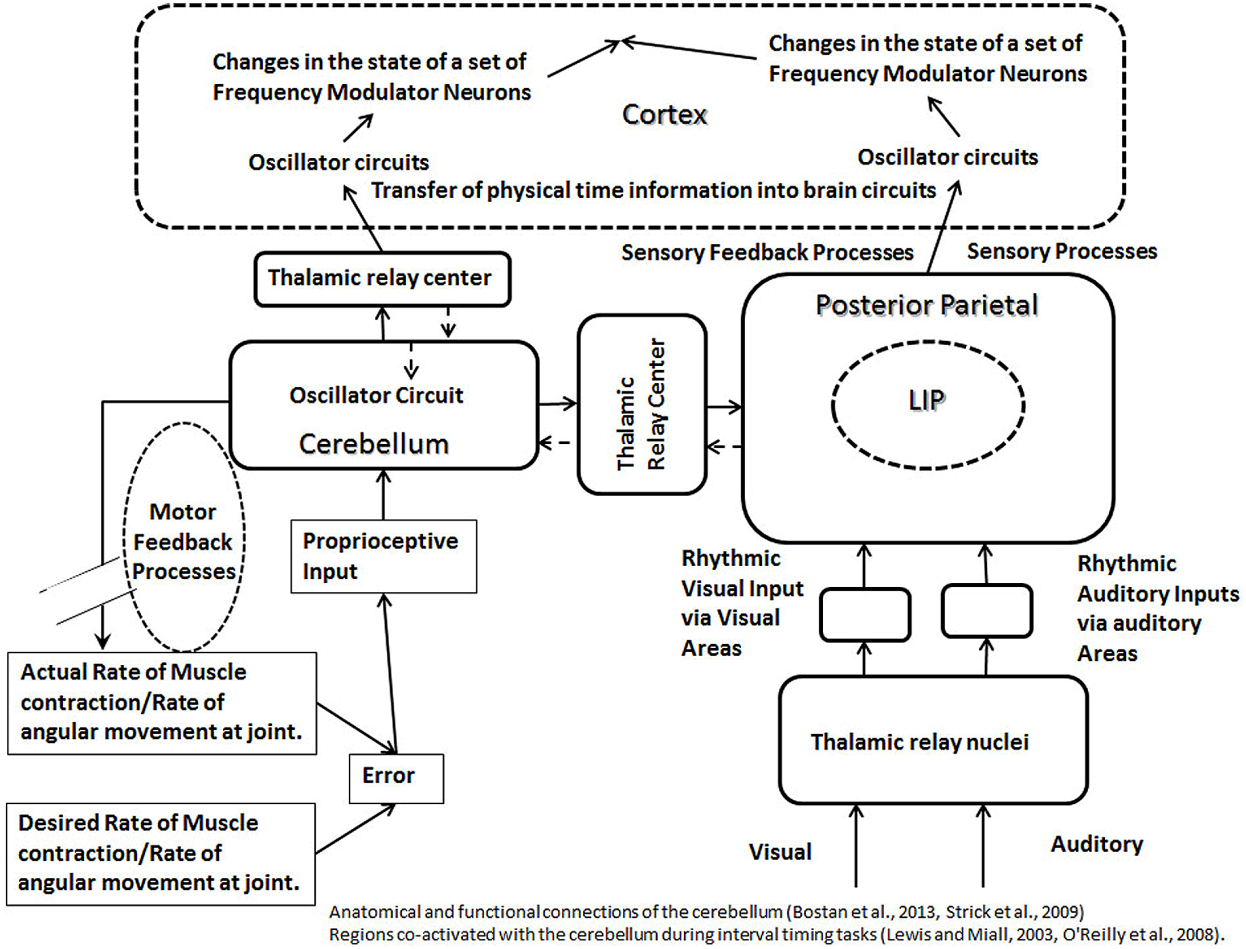
**The schematic representation of calibration modules, formed by the cerebellum (left) and posterior parietal cortex (right).** The oscillator circuits in the cerebellum and the cortex are connected to each other via the thalamic relay. Feedback processes, controlling motor movements, are shown to affect oscillator mechanism in the cerebellum. A common type of the feedback loop in the motor control involves inputs from proprioceptive pathways and outputs from motor neurons. The error produced during a motor task will be measured with the help of proprioceptive inputs, and the error correction will be accomplished by the motor output. The feedback process controlling motor movements affect the oscillators in the cerebellum and cortex, which are connected to each other via a binding property, producing synchronous changes at both levels. This helps to represent physical time intervals in circuits in the brain during an interaction with external physical event or surroundings. The irregular sensory evoked activities as well as the sensory feedback processes (in posterior parietal lobe, shown on right) will produce irregular changes in the rhythm of oscillator circuits, synchronous with the external events, helping to label the external activity on a physical time scale within circuits in the brain. Sources of the oscillator activity in the cerebellum (as discussed in the main text) include proprioceptive inputs and pacemaker neurons. Sources of the oscillator activity in the posterior parietal cortex (as discussed in the main text) are the proprioceptive inputs and polysensory processing. Different regions of the cortex receive projections from the cerebellum via thalamic relay (indicated by solid arrows), which allow cerebellum to engage with different regions of the cortex in a modular fashion during a clock mechanism. The broken arrows indicate that the reciprocal connections entering the cerebellum are less developed compared to the outgoing connections from the cerebellum.

An important function of the cerebellum is to produce a smooth control of the motor movements, which suggests its involvement in the feedback processes controlling the motor movements. Since an important role of a feedback mechanism is to maintain the normal range of a function, its failure in control of motor movement will produce an increase in the range of a movement. Consistent with the preceding, a past study has shown a four to fivefold increase in the range (representing 95% of throws) of finger opening and release times in skilled overarm throwing in patients with cerebellar lesions (Timmann et al., 1999). Furthermore, the role of the feedback processes, controlling precise movements, in transferring information about the external time into the neuronal circuits in the brain can be deduced from the discussion of an example of a cricket fielder catching a ball (**Figure 1**). To catch a ball, especially in a fast trajectory, the fielder is required to execute a complex set of temporally precise motor movements, which would recruit feedback mechanisms in the cerebellum. Missing the ball’s trajectory, even by a few fractions of a second, will result in a failure to execute the task. In other words, the feedback processes must tightly couple the motor actions to the requirements of the task parameters, such as the speed of a ball. The speed (ΔDistance/ΔDuration) of a ball contains information about time in an external three-dimensional surrounding. Therefore, the successful execution of a task will result in the transfer of information about “physical time intervals,” such as those based on the speed of external objects, into the neuronal circuits in the brain. Since most changes needed for the smooth control of motor movements occur over sub-second durations, it indicates that the cerebellum will play a role in representing shorter time durations. Consistent with this expectation, it has been shown that the brain uses networks involving the cerebellum for tasks involving shorter intervals (below 1 s; Breukelaar and Dalrymple-Alford, 1999; Ivry and Spencer, 2004; Jantzen et al., 2004). Another study showed adverse effects of the cerebellar lesions on the temporal estimation, reproduction, and production tasks (Gooch et al., 2010). Cerebellar lesions in the same study were also associated with an increase in the variability of temporal tasks (Gooch et al., 2010). The increase in the variability in temporal tasks suggests a disruption of feedback processes. Based on the preceding discussion, I propose that the feedback processes occurring in the cerebellum provide a binding property between the oscillators in the cerebellum and the cortical areas, which is responsible for maintaining the accuracy of neuronal timing mechanisms in the cortex. This binding property, present between oscillators in the cerebellum and cortical circuits, is due to the synchronous effects of feedback processes, which leads to the calibration of timing mechanisms in the cortex (**Figure 5**). The binding property is likely to be a synchronous, but a temporally sharp change at the level of the cerebellar and the cortical oscillator circuits, produced by feedback processes controlling motor movements, matching the temporal parameters of the external task. The sharp synchronous changes will help in defining or focusing temporally sharp points on time axis within different circuits in the cortex and cerebellum in synchronicity with the external temporal events, thus, transferring the external temporal information into the circuits in the brain. It is also posited that there is a direct serial connection between oscillators in the cerebellum and the cortical areas, which would be required for the flow of information between oscillators in the cerebellum and the cortical areas. Note that the flow of external physical time information from the cerebellum to the cortical areas (**Figure 5**) would require that the neural temporal units maintain their regular interval during their transmission. The pacemaker activity has been observed *in vivo* and *in vitro* in deep cerebellar nuclei (Jahnsen, 1986; Raman et al., 2000), which may represent the source of temporal units being transmitted to various clock mechanisms of the brain. Furthermore, any frequency modulation during the flow of information from an oscillator in the cerebellum to an oscillator in a cortical area is likely to disrupt the proposed binding property. A review of recent literature also finds that the output from the cerebellum projects to multiple nonmotor areas in the prefrontal and posterior parietal cortex (Strick et al., 2009), the prefrontal and posterior parietal cor providing the anatomical substrate for the binding property between the cerebellar and the cortical nonmotor circuits. The binding property can be confirmed by recording correlated activities in the nonmotor areas of the cortex and the cerebellum during motor movements.

It is argued above that external time information is represented within the neuronal circuits with the help of feedback processes, responsible for the tight coupling between the task execution and its temporal requirements. This leads us to the next question: which neurophysiological parameters help in coding the physical time as a result of the successful execution of a task? The “frequency” of neuronal firing is a good candidate for this role. Changes of activities such as the rate of muscle contraction or angular change at a joint mostly result from changes in the frequency of firing of motor neurons. Increased rate of a change in motor movements would represent shorter time durations, as the rate of change is inversely related to the time duration of occurrence of a defined change. Accordingly, a higher frequency of a neuronal activity in the nervous system will encode a shorter time duration, over which a defined activity occurs. Furthermore, as an increase in the activity of motor neurons is commonly accompanied by a general increase in the rate of the firing of neurons in various upstream circuits, a proportional increase will be seen in neuronal oscillators in the cortical and cerebellar circuits. An increased activity of oscillator circuit, according to various models, such as recurrent feedback excitation (Gavornik and Shouval, 2011), and facilitation type of short-term plasticity (Regehr, 2012) is likely to enhance the rate of the change of the activity of the proposed FM neurons. The preceding claim is supported by a study, which reported an increased rate of the change in the activity of neurons, undergoing positive modulation, when animals reported shorter anticipatory time intervals in motor tasks (Lebedev et al., 2008).

## ROLE OF SENSORY PROCESSES IN THE CALIBRATION MECHANISM

The successful execution of motor tasks, emphasized above for their role in timing mechanisms, also depends on various sensory functions, for example, cortical representations of the spatial coordinates of the surroundings, spatial coordinates of auditory stimuli, eye-centric, and body-centric visual fields. LIP area in the posterior parietal cortex area has been shown to contain body centric; and its adjacent area 7a is shown to contain world-centric representations of visual field (Andersen, 1997; Snyder et al., 1998). Neurons in LIP area also code auditory stimuli in eye-centric coordinates (Andersen, 1997). Various sensory representations in the parietal cortex can play role in feedback mechanisms in sensory processes, which can serve to calibrate oscillators in a manner discussed above for the motor processes (**Figure 5**). Consistent with the proposed role in the calibration of oscillator circuits, the left parietal lobe has been shown to be associated with the perceptual and motor temporal predictability (Coull et al., 2011). Along these lines, it is noteworthy that areas in the posterior parietal cortex, including LIP, receive proprioceptive inputs directly from the relay nuclei in the thalamus (Prevosto et al., 2011), providing a tonic input to the posterior parietal cortex, resulting from the regular bursts from the stimulation of proprioceptors, which could serve as an oscillator. A significant number of di-synaptic connections from the spinal cord dorsal column nuclei project to the posterior parietal cortex, which are the shortest possible sensory pathways to the posterior parietal cortex, and hence the most direct method of the delivery of proprioceptive inputs (Prevosto et al., 2011). Thus, the posterior parietal cortex is likely to play the role, alongside with the cerebellum, as the oscillator module, helping in the calibration in the proposed modular clock model (**Figure 5**).

Although, the role of sensory processes in transferring external temporal information via direct interaction has been emphasized, it has been argued earlier that we are able to anticipate changes in events that we cannot imitate, such as lashing waves or other inanimate events (Schubotz, 2007). The ability to anticipate can also help us in internalizing the temporal information from external events, such as falling leaves during fall, stones rolling down a mountain slope or movements of vehicles in complex traffic situations. Such ability will help in internalizing temporal information from the external events without a task execution.

The representation of external time into the neuronal circuits can be achieved by rhythmic external activities producing sensory stimulation, such as waves lashing a beach. Rhythmic external physical events, such as the waves lashing shores at a regular rate, will help to encode information about time represented within the periodicity of sounds and sights of lashing waves. This information would calibrate the oscillator activities within circuits processing rhythmic sensory inputs by directly or indirectly influencing their activities. A simple example of a periodicity coding time information will be the activation of an auditory neuron at two time points by consecutive single sound tones. In this example, the time interval between two sound tones is represented internally in the neuronal circuits by the time interval separating two evoked activities in auditory areas. In addition to providing information about the temporal periodicity, the periodic sensory inputs can modify or become an oscillator module for a neuronal clock mechanism.

Non-regular external sensory events also appear likely to play a role in coding the temporal information in neuronal circuits by producing evoked activities at irregular intervals resulting in simultaneous sharp changes in the firing activity at variable intervals, enabling the binding between different sensory circuits, similar to the effect of feedback processes during a motor activity. Such temporally non-regular events may include the sight of an object going up in the air to fall down. Temporally non-regular event can produce synchronous evoked activities in areas of cortex with different representation of same sensory modality leading a binding of oscillators in different parts of cortex, which could play a role in the calibration of timing mechanisms. A certain kind of activity may be more likely to undergo primary processing in one or the other visual field. For example, various characteristics of a speeding car will be analyzed best by organizing the visual information in the world-centric representation in the parietal cortex. In comparison, a cricket ball being sought by a running fielder would be processed best by retrieving information from the head-centric cortical representation. Accordingly, the timing mechanism for ball movement in the head-centric area of the parietal cortex will calibrate the timing mechanism in the world-centric area 7a by virtue of the binding property, enabled by synchronously evoked activities in both cortical representations. In addition, the multisensory processing circuits in the posterior parietal cortex (Andersen, 1997), may also mediate the binding of oscillator circuits of different modalities, resulting in the calibration of the timing circuits controlling multimodal tasks.

## THE ROLE OF BASAL GANGLIA IN THE MODULAR CLOCK MECHANISM

**FIGURE 6:**
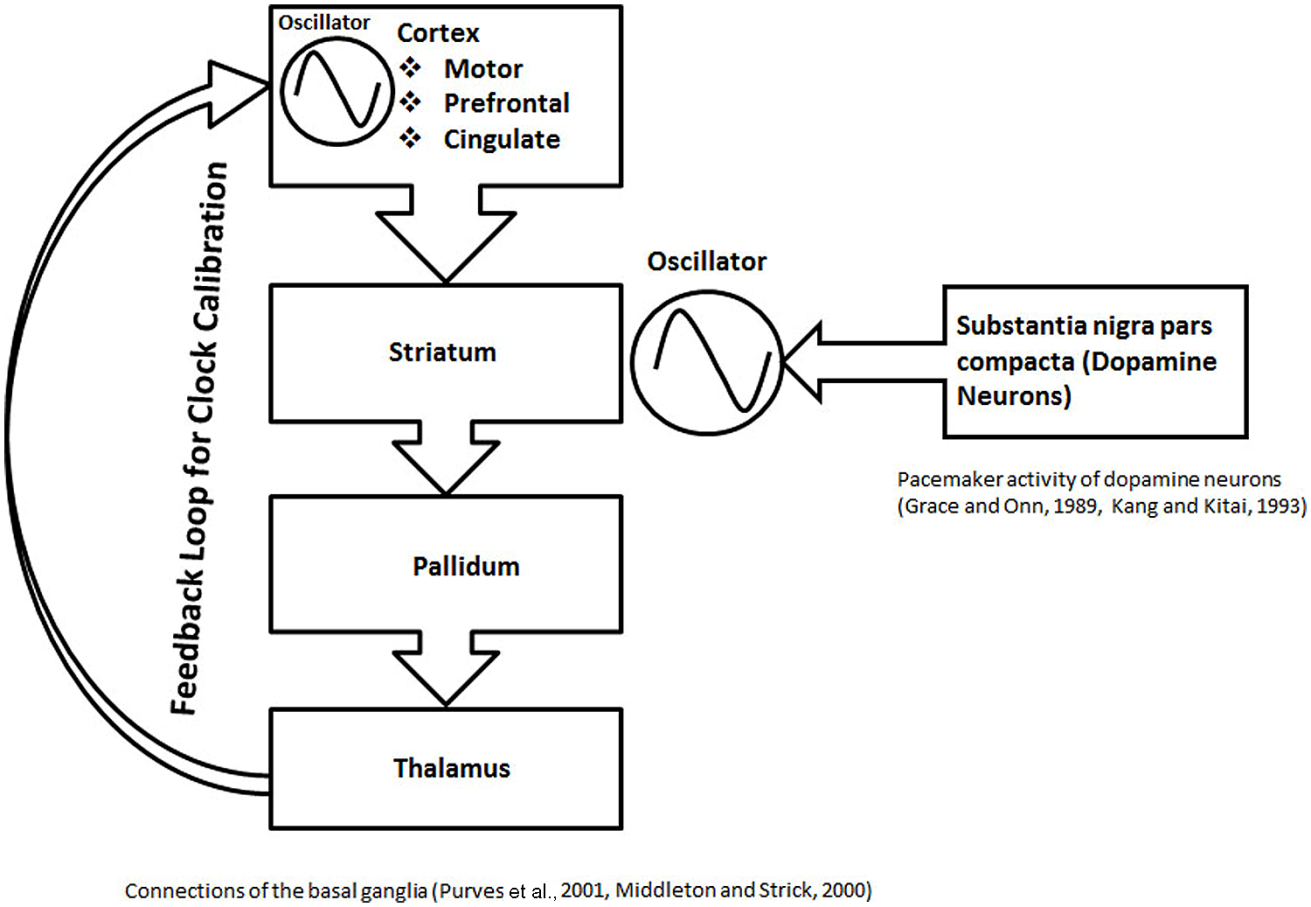
**The schematic representation of basal ganglia structures in formation of a proposed calibration module.** In this schematic, the role of the basal ganglia circuit in interval timing is illustrated in the context of the current modular clock model. An anatomical loop, formed between the basal ganglia structures, thalamus, and different cortical areas is depicted. The anatomical loop is proposed to serve as a substrate for feedback mechanisms. The striatum, which contains medium spiny neurons with stable UP and DOWN states, receives dopamine inputs from the nigral neurons with pacemaker activity. The rhythmic input of dopamine can help in the maintenance of the oscillations, resulting from the stable UP and DOWN states of the neurons in the striatum. The oscillations in loop are calibrated by the feedback processes proposed within the anatomical loop formed by the basal ganglia structures. The role of the feedback mechanism is also suggested by other types of evidence, discussed in the main text. The calibrated oscillation activity from the anatomical loop, formed by the basal ganglia structures can project to various parts of brain in a modular fashion to participate in different clock mechanisms.

Past animal studies and clinical data have implicated the basal ganglia and nigrostriatal circuits in interval timing tasks. One study showed that Parkinson’s disease subjects (withdrawn from levodopa therapy for 12–24 h) under-estimated time intervals in a verbal estimation task and over-produced time intervals, suggesting the role of dopamine and nigrostriatal circuits in an “internal clock”(Pastor et al., 1992). Another study with subjects suffering from Parkinson’s disease found impaired discrimination of time intervals in 1 s range, without impairment in the 12–42 s range (Riesen and Schnider, 2001). In contrast to the human studies, the animal studies present a more robust evidence of a substantial role of the dopamine and nigrostriatal circuit in the timing mechanisms (Coull et al., 2011). A theory to explain the role of the basal ganglia in interval timing, based on the coincidence detection was proposed earlier (Matell et al., 2003). With the help of a discussion of recent scientific literature below, I argue that the basal ganglia may play role as an oscillator module, helping in the calibration of the modular clocks (**Figure 6**).

Striatum forms part of multisynaptic, cortical–striatal–pallidal–thalamic–cortical loops (**Figure 6**), which are responsible for various functions depending on the cortical area involved, They include the motor movements (motor cortex), planning and working memory (prefrontal cortex), and limbic functions (cingulate cortex; Middleton and Strick, 2000; Purves et al., 2001). An *in vivo* recording of the medium spiny neurons, which are the main computational units in the striatum, has shown two states, an UP state (a depolarized state) and a DOWN state (a hyperpolarized state; Murer et al., 2002). Action potentials can only fire during UP state, driven by the excitatory cortical inputs (Murer et al., 2002). Thus, it is argued that the regular spaced UP states associated with regular bursts of action potentials will serve as an oscillator in the modular clock. In addition, the pacemaker activity of nigrostriatal neurons (Grace and Onn, 1989; Kang and Kitai, 1993), will release fluctuating quantities of dopamine, which would help in modulating oscillator pattern, produced by the firing of the striatal medium spiny neurons. The multisynaptic, cortical–striatal–pallidal–thalamic–cortical circuit is an anatomical loop which connects the cortex, basal ganglia and thalamus. Since many negative feedback processes are represented by loop-like pathways, cortical–striatal–pallidal–thalamic–cortical circuit is a likely anatomical substrate for a feedback loop with a role in cortical information processing. Although it is not clear if the thalamic connections return to the same cortical region, it is a possibility that an anatomical loop may be completed by the cortical association fibers. On the basis of the above discussion, combined with the evidence from different studies discussed below, I posit that cortical–striatal–pallidal–thalamic–cortical circuit would serve as the anatomical substrate for the feedback mechanisms to play a role in the calibration of the proposed basal ganglia clock (**Figure 6**).

A study that simultaneously recorded single neuron activities from the primary motor and sensory cortex together with the putamen during a visual motor task, found spatial and temporal task-related firing of neurons across these regions (Santos et al., 2014). The correlated firing behavior is consistent with the accurate transmission of information (Santos et al., 2014), which is necessary for a feedback mechanism. Another study showed coherence during the simultaneous recordings of the electrocorticogram and local field potential in the basal ganglia (Magill et al., 2004). Coherent slow waves (∼1 Hz) and spindle frequency (7–12 Hz) oscillations recorded in the cortico-basal ganglia circuits switched to a coherent, medium to high-frequency (15–60 Hz) activity after sensory stimulation by pinching hindpaws at 15 s intervals with serrated forceps (Magill et al., 2004). The state-dependent coherence in the activity is consistent with information processing during a feedback mechanism. A direct involvement of the cortical–striatal–pallidal–thalamic–cortical circuit in the feedback mechanisms is further indicated by asynchronous activity after the onset of movements observed in basal ganglia during the simultaneous recording of multiple neurons in monkeys (Jaeger et al., 1995).

The nigral dopaminergic inputs to striatal spiny neurons may play a role in the feedback loops by maintaining the activity in cortical–striatal–pallidal–thalamic–cortical circuits. Accordingly, the loss of nigrostriatal dopamine input to striatal circuits would lead to the disruption of the feedback mechanism (**Figure 6**). This is indicated by the increased variations in timing tasks in Parkinson’s disease (Pastor et al., 1992). Also note that the role of the basal ganglia circuit in motor control is likely to be different from its role in the calibration mechanism in the modular clock. This is indicated by a recent study, examining the effects of the bilateral high-frequency stimulation of the basal ganglia, used widely for the treatment of the motor symptoms of Parkinson’s disease, on the duration and beat based tasks of the sub-second range (Cope et al., 2014). This study found no significant effect on any perceptual timing task studied (Cope et al., 2014), which could be due to the inability of bilateral high-frequency stimulation of the basal ganglia to significantly influence the proposed feedback processes in the cortical–striatal–pallidal–thalamic–cortical circuits.

## THE ROLE OF CEREBELLAR CIRCUITS IN THE MODULAR CLOCK MECHANISM

Past studies have shown that the brain uses networks involving the cerebellum for tasks involving sub-second intervals (Breukelaar and Dalrymple-Alford, 1999; Ivry and Spencer, 2004; Jantzen et al., 2004). In addition, the lesions of the cerebellum increase variability on various timing tasks, which include the temporal estimation, reproduction, and production tasks (Gooch et al., 2010). The above is consistent with the proposed role of the cerebellum as a module responsible for the calibration of neuronal clocks, for timing intervals in the sub-second range. It is also argued that the binding between oscillators in the cerebellum and cortical areas, as a result of feedback processes controlling motor movements, is responsible for the calibration of the modular clocks in cortical areas (**Figure 5**). The pacemaker activity has been observed *in vivo* and *in vitro* in deep cerebellar nuclei, forming the main output regions of the cerebellum (Jahnsen, 1986; Raman et al., 2000), which may represent an oscillator, projecting to another oscillator circuit in the cortical areas. An inter-connection or binding between oscillators would help in transferring information about physical time from the cerebellar circuits to the cortex during tasks requiring interaction with the external world (**Figure 5**). Furthermore, the changes that occur during a motor control are commonly in the sub-second range, which supports the role of the cerebellum in the sub-second timing.

## PROCESSING TIME INTERVALS WITHOUT ANY EXTERNAL TASKS OR CUES

Since the processing of time intervals, in the absence of cues, does not involve any specific tasks, the calibration of clock mechanism will not be accomplished by a successful task completion. Instead, the mechanism for interval timing in the absence of cues would be linked to the sensory perception of the passage of time. This is based on the observed relationship between the sense of passage of time and the subjective estimation of time intervals. For example, a subjective report of the fast passage of time is often associated with the over-estimation of time intervals. The sensory perception of the flow of time is likely to be a consequence of the sensory detection of the temporal order, when there is a minimum temporal separation between adjacent multimodal or monomodal stimuli, for example by 20–30 s for auditory stimuli (Poppel, 1994, 1997; Pastore and Farrington, 1996). During the conscious state, there is a task-independent processing of multimodal stimuli, such as auditory, proprioceptive and visual, associated with the perception of the flow of time in the posterior parietal cortex, which will generate oscillator-type activities. I, furthermore, posit that the task-independent polysensory processing will act as an oscillator in the modular clock in the absence of external cues. This agrees with a study in primates (Schneider and Ghose, 2012), in which the production of inter-saccade intervals in the range of 800–1200 ms in absence of any external cues, was positively correlated with the variations of the frequency of neurons with descending activity in LIP area in the posterior parietal cortex, believed to be an important area for the polysensory processing in the brain (Andersen, 1997; Bremmer et al., 2001; Macaluso and Driver, 2001).

A potential mechanism of calibration in the absence of cues would be the feedback process based on the cortical–striatal–pallidal–thalamic–cortical loop. The anatomical evidence, supporting the role of cortical–striatal–pallidal–thalamic–cortical loop in the calibration of clock circuits comes from a study using the retrograde transneuronal transport of rabies viruses to examine the subcortical inputs to anterior intraparietal area in the posterior parietal cortex, which found labeling of a substantial number of neurons in the substantia pars reticulata (Clower et al., 2005), one of the output structures of the basal ganglia (Middleton and Strick, 2000). Another potential method of the calibration of clocks in a cue-independent mode is a pre-stored pattern of activity in the premotor region, which is commonly co-activated with the inferior parietal region in fMRI studies during timing tasks, without specific instructions to time (Coull and Nobre, 2008).

## SENSORY PROCESSING IS COUPLED WITH THE PERCEPTION OF TIME INTERVALS

Sensory experience of external events involves simultaneity of perception of different modalities, which results from the polysensory processing in the parietal cortex, involving gain modulation (Andersen, 1997). Since many timing tasks are multimodal in nature, the polysensory processing is likely to help in the transfer of the external time information into the nervous system. The involvement of polysensory processing in timing mechanisms comes from a study, in which subjects compared the interval between stimuli of same (monomodal) or different (polymodal) modalities with a standard time interval (Harrington et al., 2011). During a multimodal timing task, comparing time interval between auditory and visual sensory stimuli, there was increased activation of the parietal frontal network (Harrington et al., 2011). In contrast, the monomodal timing task showed greater activation of the striatum (Harrington et al., 2011). This suggests that timing mechanism, involving cues of different sensory modalities, is likely to be a part of polysensory processing circuits in the posterior parietal lobe. Thus, it is not surprising that networks in the brain, involved with the measurement of time intervals in most studies, include the posterior parietal region (Amino et al., 2001), which contains several important polysensory processing areas, as well as various representations of the visual and auditory fields (Andersen, 1997). Functional imaging studies have shown that the polysensory processing areas in humans and primates are present in the superior temporal sulcus and intra parietal sulcus (Bremmer et al., 2001; Macaluso and Driver, 2001). PET studies have shown that the tasks requiring the detection of the visual auditory asynchrony activate a large network in the brain that includes the posterior parietal, prefrontal, and cerebellar areas (Bushara et al., 2001), which suggests a relationship between the polysensory and temporal processing. Moreover, past studies have shown that the visual and auditory response fields often overlap in the parietal cortex, and also show gain modulation (Andersen, 1997). Gain modulation, which produces a non-linear effect on the perception of another modality, is likely to play a role in the multimodal timing tasks.

## SYNOPSIS OF THE PROPOSED MODULAR CLOCK MODEL

The current model postulates “neuronal temporal units,” which represent the external time information in neuronal clocks in the brain. “Neuronal temporal unit,” defined as the interval between two adjacent spikes or spike bursts resulting from the regular activity of neuronal oscillators, is calibrated, so that it represents physical time accurately within the neuronal circuits. The module containing the neuronal oscillator is responsible for the calibration of the clock. The mechanisms for the calibration of neuronal oscillators include (a) feedback processes involving motor movements, (b) binding between different circuits that result from the synchronous effects of the feedback processes or sensory processes, and (c) input of temporal periodicity resulting from a regular sensory stimulation. The calibration of the neuronal clocks involves a successful transfer of the temporal information from the external physical events into the neuronal circuits, via the successful execution of various tasks or functions. The transfer of external physical time information may sometimes involve two levels of oscillator circuits. For example, during motor tasks, there is a proposed involvement of two oscillators, which help in the transfer of external physical time information. The first-level oscillator is present in the calibration module, such as the cerebellum (**Figure 5**). The second-level oscillator is inbuilt within various circuits in the brain. The second-level oscillator (inbuilt) is in a binding state with oscillators in one of several calibration modules. The ability of an inbuilt oscillator to bind to the oscillators in different calibration modules is responsible for the modular nature of the clock. The effect of feedback and sensory processes on the oscillator in the calibration module would produce synchronous effects on various inbuilt oscillators due to the binding, which is proposed to lead to the representation of physical time in various brain circuits. Note that the external information can be also imported into neuronal circuits without a direct interaction with the external world (Schubotz, 2007).

No single algorithm for the processing of the neuronal temporal units is proposed in the current model. As discussed earlier, the output of oscillator circuits is likely to be processed by several possible mechanisms to produce a ramping or a decaying-type modulation in the activity of FM neurons. The frequency or the rate of the change of the frequency, resulting from the modulation in the activity of FM neurons, codes the time intervals in the brain circuits. This is in line with the prevailing view that many mechanisms may be responsible for computing time intervals (Lewis and Miall, 2003; Wittmann, 2009; Teki et al., 2011a). Different networks of brain are found to be active in different interval timing tasks depending on nature, motor vs. perceptual, or the duration sub-second vs. supra-second (Wiener et al., 2010a). According to the current model, the differences in the mechanism of interval timing in different tasks result from the modular nature of the proposed clock.

### DIFFERENTIAL ACTIVATION OF NETWORKS IN THE BRAIN IN DIFFERENT TIMING TASKS

Timing tasks are classified as (a) explicit interval timing, when subjects report duration by speaking, or performing delayed or prolonged motor task; and (b) implicit interval timing, when regular pattern of sensory stimuli or motor responses is used to predict non-temporal parameters, such as, velocity of object trying to land (Coull et al., 2011). Explicit timing is commonly associated with the activation of the basal ganglia, and the co-activation of the prefrontal, premotor, and cerebellar areas seen in more context-dependent tasks (Coull and Nobre, 2008). This is consistent with the current proposal for the role of the basal ganglia in the modular clock for a cue-independent timing. On the other hand, an implicit perceptual timing task recruits the inferior parietal and the premotor area (Coull and Nobre, 2008), suggesting the importance of the visual and spatial information processed in the posterior parietal cortex. Another fMRI study had shown the activation of the left cerebellar hemisphere in addition to the right hemispheric pre-supplementary motor area, frontal pole, and inferior parietal cortex in an explicit interval timing task requiring the discrimination of sub second intervals (Lewis and Miall, 2003). Also, a meta-analysis of neuroimaging studies showed activation of the left inferior parietal cortex in implicit timing tasks (Wiener et al., 2010b). Furthermore, a fMRI study, involving the direction (spatial) and velocity (spatial and temporal) judgment tasks, showed co-activation with parietal regions only during the velocity (spatial and temporal) judgment task, but not during direction only task (O’Reilly et al., 2008), which underscores the “engagement” of the cerebellum only during temporal tasks. Anatomical studies have shown that the cerebellar outputs also target multiple nonmotor areas, especially the prefrontal and posterior parietal cortex, implicated in interval timing (Strick et al., 2009; Bostan et al., 2013), which provides the anatomical basis for the co-activation of cortical regions with the cerebellum during different timing tasks. The differential activation of brain networks during different timing tasks suggests the modular nature of the neuronal clocks, which is consistent with the currently proposed clock model (**Figure 5**).

### MODULAR NATURE OF THE NEURONAL CLOCK MODEL AND ITS IMPLICATION

Consistent with the modular nature of the neuronal clocks, I propose that one or several calibration mechanisms would serve in a single clock mechanism. This feature of the modular clock model introduces redundancy in the clock mechanism of the brain. In individuals with the lesions of the cerebellum, other calibration mechanisms, mainly sensory, will take over the function of calibrating neuronal clocks. Since mechanisms based on the sensory processes calibrate or internalize time intervals of longer durations in comparison to the motor processes, the cerebellar lesions will result in greater variations during the processing of temporal intervals. Evidence for the preceding is provided by a study, which showed that the cerebellar lesions disrupted the precise timing, indicated by increased variations (Timmann et al., 1999; Ivry and Spencer, 2004). Furthermore, the multiplicity of calibration mechanisms and multiple oscillator circuits built inside various circuits spread across the brain would explain why no neurological or psychiatric illness is characterized by a complete deficiency of the perception of time intervals.

### MULTIPLE CALIBRATION MODULES AND THE IMPLICATIONS

The discussion of different circuits in the current manuscript also indicates that the posterior parietal areas, pre-motor area and basal ganglia circuit (cortical–striatal–pallidal–thalamic–cortical circuits) also are likely to serve as calibration modules in addition to the cerebellum. An earlier fMRI study had found significant activations in a cerebellar network during absolute, duration-based timing and a striato–thalamo–cortical network during relative, beat-based timing (Teki et al., 2011b). Since processing the absolute duration will require input of physical time into brain circuits, the role of cerebellum in processing absolute durations is consistent with its role in the motor tasks, which help to produce representation of the physical time in the brain circuits. However, the proposed cortical-striatal-pallidal-thalamic-cortical circuits are closed loops, with no likely direct interaction with the external events. Accordingly, the internal homeostasis of cortical information processing may be the primary function of the feedback processes in the cortical-striatal-pallidal-thalamic-cortical circuits. With no direct representation of physical time, basal ganglia circuits may mainly play role in the beat-based timing, which is relative to a rhythm. This is consistent with activation of basal ganglia during beat-based timing tasks (Teki et al., 2011b).

### SUB-SECOND VS. SUPRA-SECOND INTERVAL TIMING

While the cerebellum is implicated in the sub-second interval timing tasks, the imaging and clinical data implicate the basal ganglia in both, the sub- as well as the supra-second interval timing tasks (Malapani et al., 1998; Wiener et al., 2010a). A meta-analysis study showed that different networks are activated in sub-second vs. supra-second timing tasks (Wiener et al., 2010a). Many of the differences reported in this study are likely to be related to the differential activation of the second module, associated with various tasks. Furthermore, because of the modular nature of the proposed neuronal clock, a particular calibration module, for example, the cerebellum may become differentially co-activated within different networks of the brain during various sub-second tasks. In an example of the second module activation, sub-second perceptual tasks demonstrate the highest activation likelihood in the left inferior frontal gyrus, which is an important cortical area for the language production (Wiener et al., 2010a).

### A COMMON LINK BETWEEN THE SCALAR PROPERTY OF THE INTERVAL TIMING AND INCREASED VARIATIONS ON TIMING TASKS IN CEREBELLAR LESIONS?

The scalar property of the interval timing, that is, the variations in interval timing increases with its increasing duration (Piras and Coull, 2011), may be linked empirically to engaging different calibration mechanisms in tasks of different durations (**Figure 5**). This can be explained with the help of an example of a stochastic process, which assembles anticipatory time intervals with a random number of temporal units of a standard length, to produce the best match with a task requirement. During the production of time intervals with temporal units of a longer standard length by such process, one is likely to find an increase in variations. This type of random process may also explain the presence of greater variations in the production of time intervals of greater length, when a modular clock uses a different calibration mechanism, such as the sensory processes, instead of the cerebellar mechanisms, which is more suitable for the shorter time intervals. For similar reasons, the cerebellar lesion leads to an increase in the variability of temporal tasks (Gooch et al., 2010). Following the cerebellar lesions, other calibration modules would be responsible for calibration, which help in creating representations of longer intervals in the brain. This would result in increase in the variations, as argued above with the help of a random assembly process.

### ABNORMAL INTERVAL TIMING IN SCHIZOPHRENIA

In a recently published study, schizophrenic patients were found to underestimate anticipatory time intervals during the tracking of the movement of visual stimuli under cognitively challenging conditions (Peterburs et al., 2013). Common basis for the cognitive defect and impairment of interval timing in schizophrenia can be explained with the help of this model. The likely target for common pathological changes would be various circuits controlling cognitive functions with inbuilt clocks, for example, cortical–striatal–pallidal–thalamic–cortical circuit. Damage to above circuits would contribute to cognitive symptoms, in addition to the impairment of the interval timing functions. Furthermore, a disruption of the general feedback processes involved in motor control, playing a role in the calibration of inbuilt clocks in the proposed model, would explain a high prevalence rate of motor symptoms observed in schizophrenia (Walther and Strik, 2012). A high prevalence rate of the motor symptoms in schizophrenia suggests an involvement of the cerebellum. Anatomical and physiological studies have shown that the cerebellum projects to myriad neocortical areas, such as the prefrontal and parietal cortex, and contributes to the cognition and visuospatial reasoning (Strick et al., 2009; Bostan et al., 2013). Therefore, the proposed inbuilt clocks, calibrated by the cerebellar processes, could be involved in schizophrenia, producing cognitive as well as motor symptoms.

### FUTURE DIRECTIONS

Emerging evidence indicates that the “correlated noise” between neurons and its structure help pools of neurons in guiding their behavior (Nienborg and Cumming, 2010). Although the present model does not to assign a clear role to the “correlated noise,” it may play an important role in decoding the activities of FM neurons, helping in the processing of different time intervals, and the decision-making processes. Mounting evidence also suggests that stochastic processes play an important role in information processing in the brain (Xing et al., 2012). Correlated noise and population dynamics could play role at different levels in this model, which include (a) the feedback processes influencing the oscillator circuits, (b) modulation of FM neuron activity by oscillator circuits, and (c) decoding of activity of FM neurons by circuits involved in various tasks. Future study of the proposed modular clock mechanisms would also address the role of the population dynamics of neurons or synaptic connections, or both in complex modulations seen in various timing tasks.

## Conflict of Interest Statement

The author declares that the research was conducted in the absence of any commercial or financial relationships that could be construed as a potential conflict of interest.
